# The quest for personalized B-cell depletion therapy in rheumatic disease

**DOI:** 10.1186/ar4595

**Published:** 2014-06-26

**Authors:** Kiran Nistala, Claudia Mauri

**Affiliations:** 1 Centre for Rheumatology, Division of Medicine, University College London, The Rayne Building, 4th Floor, Room 424, 5 University Street, London WC1E 6JF, UK

## Abstract

Although B cell depletion therapy (BCDT) is now a well-accepted therapeutic option in autoimmune rheumatic disease, a significant proportion of patients remain resistant to therapy. .19pt?>A more challenging clinical problem is the high rate of relapse after B cell reconstitution, as well as the difficulty in predicting the exact timing of that relapse. In this article, we consider the immunological mechanisms that may account for the heterogeneity of clinical response to BCDT. Understanding how BCDT alters the balance between different B cell subsets, some pathogenic and some regulatory, may help us correctly target BCDT to the right patients, and thereby improve treatment responses in rheumatic disease.

## 

The identification of autoantibodies in the serum of patients with rheumatic disease was one of the landmark studies that placed B cells at the heart of research into the pathogenesis of autoimmune disease. It is now clear that B cells contribute to autoimmunity by a range of mechanisms, both directly through the secretion of inflammatory cytokines [1] and indirectly by antigen presentation and co-stimulation to activate autoreactive T cells. However, it was only at the beginning of the last decade that attention finally turned to B cells as a potential target that may ameliorate autoimmune rheumatic disease.

## B-cell depletion therapy in rheumatic disease

Randomized controlled trials (RCTs) of the anti-CD20 antibody rituximab provided the first evidence that B-cell depletion therapy (BCDT) reduces disease activity in rheumatoid arthritis (RA). In systemic lupus erythematosus (SLE), BCDT was found to be highly effective in routine clinical practice and open studies, and so it was surprising that two RCTs of BCDT in SLE failed to meet their primary end point [2,3]. This may relate, at least in part, to issues of patient selection and trial design, such as the use of concomitant high-dose corticosteroids (reviewed in [4]). B-cell effector function may be important for predicting response to BCDT, as anti-CD20 therapy was successful in an animal model of multiple sclerosis but only if B cells secreting IL-6 were contributing to pathology [5]. Whether this is true of autoimmune rheumatic disease is still unclear. Certainly in clinical practice, some RA patients resistant to BCDT still respond to anti-IL-6 blockade, suggesting that alternative sources of IL-6 may be important in disease persistence (personal communication, David Isenberg, University College London).

A further problem in assessing BCDT is that successful depletion is defined by circulating total B-cell counts and this disregards the diversity of B-cell phenotype, function, and compartmentalization. A pooled analysis of more than 800 patients from different RCTs indicated that plasmablast markers are useful in identifying a subgroup of non-responders in RA [6]. Also, long-lived plasma cells that express low levels of CD20 and reside within the bone marrow and spleen may further contribute to persist disease as seen in the case of patients with immune thrombocytopenia treated with rituximab [7]. In SLE, increased serum levels of the B cell-activating factor following repeated rituximab therapy were associated with elevated anti-double-stranded-DNA antibodies and disease flare [8]. Taken together, these studies suggest that continued activity of plasmablasts/plasma cells may be one explanation for the persistence of disease following BCDT.

As well as predicting resistance to initial BCDT, a further clinical challenge is to prevent relapse of disease in those patients who have undergone remission. In some patients, relapse closely follows B-cell repopulation, whereas in others relapse can be delayed for years [9,10]. These data suggest that the functional characteristics of the emergent B-cell population may be more important than the simple fact of reconstitution.

## Understanding B-cell heterogeneity – the role of regulatory B cells

Over the last decade, our group, and others, have identified a novel subset of B cells with an immunoregulatory role rather than one of pathogen clearance. These regulatory B (B_reg_) cells function in an IL-10-dependent manner [11] to suppress inflammatory T-cell responses and induce regulatory T cells, leading to the suppression of arthritis and lupus in mouse models [11,12]. We have recently discovered, within the circulating immature B-cell compartment in humans, similar populations of cells that are the equivalent of murine B_reg_ cells. These human B_reg_ cells restrain T-cell responses *in vitro* and are numerically or functionally deficient in rituximab-naïve patients with RA and SLE [13,14]. Although B_reg_ cells express CD20 and are likely to be depleted by BCD, the effects of a reduced immune regulatory pool may be masked by the simultaneous reduction in pathogenic B cells. However, this temporary status quo is unstable and may be easily disturbed depending on which B-cell population is first to repopulate after BCDT. Given that immature cells are often the first B cells to return in circulation [15], we predict that these cells, rather than contributing to disease relapse, may in fact be regulatory and thus play an important role in maintaining immune tolerance after BCDT. Our recent data exploring the interaction between B_reg_ cells and invariant natural killer T (iNKT) cells, a rare subset of innate-like T cells with homeostatic function, support this hypothesis. In health, immature B cells promote the expansion of anti-inflammatory iNKT cells [16], whereas B cells from patients with active SLE are defective and fail to maintain iNKT cells, leading to significantly reduced iNKT cell numbers in circulation. Following BCD, patients who repopulated with immature B cells normalized iNKT cell numbers and maintained a clinical response to BCDT. These data suggest that repopulation of B cells with a regulatory phenotype may be important in maintaining clinical remission. In contrast, the repopulation with circulating memory B cells or plasmablasts has been associated with earlier relapse of disease in SLE [17].

Therefore, it is likely that the initial response to BCDT, as well as the risk of disease relapse afterwards, depends on the specific mechanisms of B-cell pathology and the balance between effector and B_reg_ cell subsets in the individual patient. To identify biomarkers that can accurately predict response to BCDT, we need a better understanding of B-cell heterogeneity and surface markers that can precisely distinguish B cells with effector or regulatory function. These results would open up the possibility of screening patients prior to BCD to assess effector/regulatory balance and see whether this better predicts treatment response. Importantly, this work offers a chance to move toward a more personalized form of BCDT, one that will hopefully reduce the risks of treatment resistance and relapse in autoimmune rheumatic disease.

## Note

This article is part of the collection ‘*Why is there persistent disease despite aggressive therapy of rheumatoid arthritis?*’, edited by Pierre Miossec. Other articles in this series can be found at http://arthritis-research.com/series/residual.

## Abbreviations

BCD: B-cell depletion; BCDT: B-cell depletion therapy; B_reg_: regulatory B; IL: interleukin; iNKT: invariant natural killer T; RA: rheumatoid arthritis; RCT: randomized controlled trial; SLE: systemic lupus erythematosus.

## Competing interests

The authors declare that they have no competing interests.
